# Supplementary material for: Automated Echocardiographic Detection of Mitral Valve Prolapse and Mitral Regurgitation with Video-based Artificial Intelligence Algorithms
Source: medRxiv. 2026 Mar 2:2026.02.26.26347229. Preprint. [Version 1] doi: 10.64898/2026.02.26.26347229 (PMC12976903; doi:10.64898/2026.02.26.26347229)
Supplement: 1 [file NIHPP2026.02.26.26347229v1-supplement-1.pdf]

medRxiv preprint doi: https://doi.org/10.64898/2026.02.26.26347229; this version posted March 2, 2026. The copyright holder for this preprint (which was not certified by peer review) is the author/funder, who has granted medRxiv a license to display the preprint in perpetuity.
It is made available under a CC-BY 4.0 International license .

Supplementary tables

Supplementary table 1: Comparison of Single-view vs. Multi-view MVP models on UCSF test set

	AUC	Sensitivity	Specificity
**A2C view model**	0.862 (0.839-0.883)	0.694 (0.644-0.743)	0.858 (0.849-0.867)
**A4C view model**	0.862 (0.840-0.881)	0.739 (0.688-0.784)	0.801 (0.791-0.812)
**PLAX view model**	0.879 (0.860-0.898)	0.752 (0.705-0.799)	0.839 (0.828-0.849)
**Multi-view model**	0.917 (0.899-0.934)	0.797 (0.754-0.841)	0.893 (0.884-0.901)
